# *Saccharomyces cerevisiae* in cancer research: modeling tumor biology and enabling drug discovery

**DOI:** 10.15698/mic2026.08.885

**Published:** 2026-08-03

**Authors:** Graziana Assalve, Paola Lunetti, Annalisa Fai, Luca Frattaruolo, Anna Rita Cappello, Alessandra Ferramosca, Vincenzo Zara

**Affiliations:** 1 Department of Experimental Medicine, University of Salento, 73100 Lecce (LE), Italy; 2 Department of Pharmacy, Health & Nutritional Sciences, University of Calabria, 87036 Rende (CS), Italy

**Keywords:** *Saccharomyces cerevisiae*, cancer, signaling pathways, metabolism, DNA repair, autophagy, drug discovery

## Abstract

Cancer is a complex disease driven by genetic, metabolic, and environmental alterations, whose investigation is often constrained by the limited tractability of mammalian systems. The budding yeast *Saccharomyces cerevisiae* has emerged as a powerful eukaryotic model to study conserved cellular processes relevant to tumor biology in a simplified and scalable context and as a versatile platform for translational and biotechnological applications. Through genetic manipulation and heterologous expression, yeast allows systematic analysis of human cancer genes and variants, providing quantitative insights into their functional impact. In parallel, yeast reproduces fundamental features of cancer cell metabolism and stress adaptation, offering a controlled system to investigate cellular responses to environmental constraints. The conservation of major DNA repair and autophagy pathways further supports the use of yeast to study genome stability and survival mechanisms in cancer. Beyond its role in basic research, *S. cerevisiae* represents a scalable platform for anticancer drug discovery, enabling systematic identification of drug targets, resistance mechanisms, and genotype-specific vulnerabilities through high-throughput and engineered strain-based approaches. Continued development of yeast platforms, together with synthetic biology, functional genomics, and advanced genomic technologies, is expected to accelerate therapeutic innovation and improve our understanding of cancer biology. This review discusses recent advances in yeast-based cancer research, highlighting the contribution of engineered yeast platforms to the investigation of oncogenic signaling, metabolic rewiring, stress adaptation, DNA repair, and autophagy, reinforcing the role of yeast at the interface between cancer research and biotechnology.

## INTRODUCTION

Cancer remains a major global health challenge, driven by complex genetic, metabolic, and environmental alterations. Despite significant advances in cancer research, the study of tumor biology and the development of effective therapeutic strategies are often limited by the complexity and low tractability of mammalian systems. These limitations have generated a growing demand for simplified, scalable, and genetically tractable platforms capable of enabling mechanistic studies and high-throughput approaches.

The budding yeast *Saccharomyces cerevisiae* has emerged as a powerful eukaryotic model system for investigating conserved cellular processes relevant to human disease 1, 2. Its genetic tractability, rapid growth, and compatibility with large-scale experimental approaches make it particularly well suited for functional genomics, pathway reconstruction, and the systematic analysis of disease-associated genetic variants 3, 4. A substantial proportion of genes involved in fundamental processes such as cell cycle regulation, DNA repair, and growth signaling are conserved between yeast and humans, enabling functional studies of human proteins in a simplified cellular context 5–7. These genes are frequently mutated or dysregulated in cancer cells, leading to uncontrolled proliferation and genomic instability, thereby underscoring the value of yeast as a powerful model system for studying conserved cancer-related processes across tumor types 8.

In addition to its genetic conservation, yeast reproduces key aspects of cancer-associated metabolic reprogramming. Similar to tumor cells, yeast can dynamically adapt its metabolism in response to environmental and nutrient fluctuations, shifting between respiratory and fermentative states to sustain proliferation 9, 10.

Yeast also provides a tractable system for studying tumor microenvironment (TME)-associated stresses relevant to tumor biology, including hypoxia, oxidative stress, and nutrient deprivation 11. Experimental growth under oxygen-limited conditions enables the investigation of cellular adaptation mechanisms that parallel those occurring within the TME.

Defects in DNA repair pathways are a hallmark of cancer and contribute to genomic instability and tumorigenesis 12. The conservation of major repair systems in yeast, including homologous recombination (HR), nucleotide excision repair (NER), and base excision repair (BER), allows their systematic dissection in a simplified genetic background 10. Likewise, the conservation of autophagy-related genes between yeast and humans enables the use of *S. cerevisiae* to investigate how cancer cells exploit this pathway to survive under stress conditions such as nutrient limitation and chemotherapy exposure 13, 14.

Collectively, the conservation of these fundamental pathways positions yeast as a powerful system for modeling cancer-associated alterations and evaluating their functional consequences in a controlled and scalable manner.

Beyond its role as a model organism, yeast also represents a powerful biotechnological platform for drug discovery 15. Engineered yeast strains expressing human oncogenes or tumor suppressor mutations enable high-throughput screening (HTS) of small molecules targeting cancer-related pathways, thereby accelerating the identification of potential therapeutic candidates 16. In addition, yeast-based systems allow functional characterization of mutations associated with drug resistance, providing insight into mechanisms of therapy failure and supporting the development of more effective anticancer strategies 10, 17.

In this review, we highlight the use of *S. cerevisiae* as a model system to bridge fundamental cancer biology with translational and therapeutic research, emphasizing how conserved features shared between yeast and human cells provide meaningful insights into oncogenic processes. We specifically focus on how yeast-based approaches have contributed to deciphering cancer-associated pathways, modeling tumor-relevant cellular stresses, and accelerating the discovery of novel therapeutic vulnerabilities. We further discuss the advantages and limitations of this approach, together with emerging perspectives on the integration of yeast models into modern cancer research pipelines, and key yeast-based discoveries that have contributed to understanding the molecular basis of tumorigenesis.

## YEAST-BASED PLATFORMS FOR MODELLING CANCER HALLMARKS

### Conserved oncogenic signaling pathways

The budding yeast *S. cerevisiae* is widely used as a eukaryotic platform to dissect conserved oncogenic signaling pathways, including RAS, PI3K, and p53 networks, as well as key tumor suppressor systems 18–20. These pathways, frequently altered in human malignancies such as lung, breast, colorectal cancers, and glioblastoma, regulate fundamental processes including cell cycle progression, genomic stability, and cellular metabolism 21–24. Because many of these signaling modules are evolutionarily conserved, yeast provides a uniquely tractable system to investigate how oncogenic mutations perturb core cellular functions and to identify vulnerabilities that may be therapeutically exploited.

**Figure 1:**
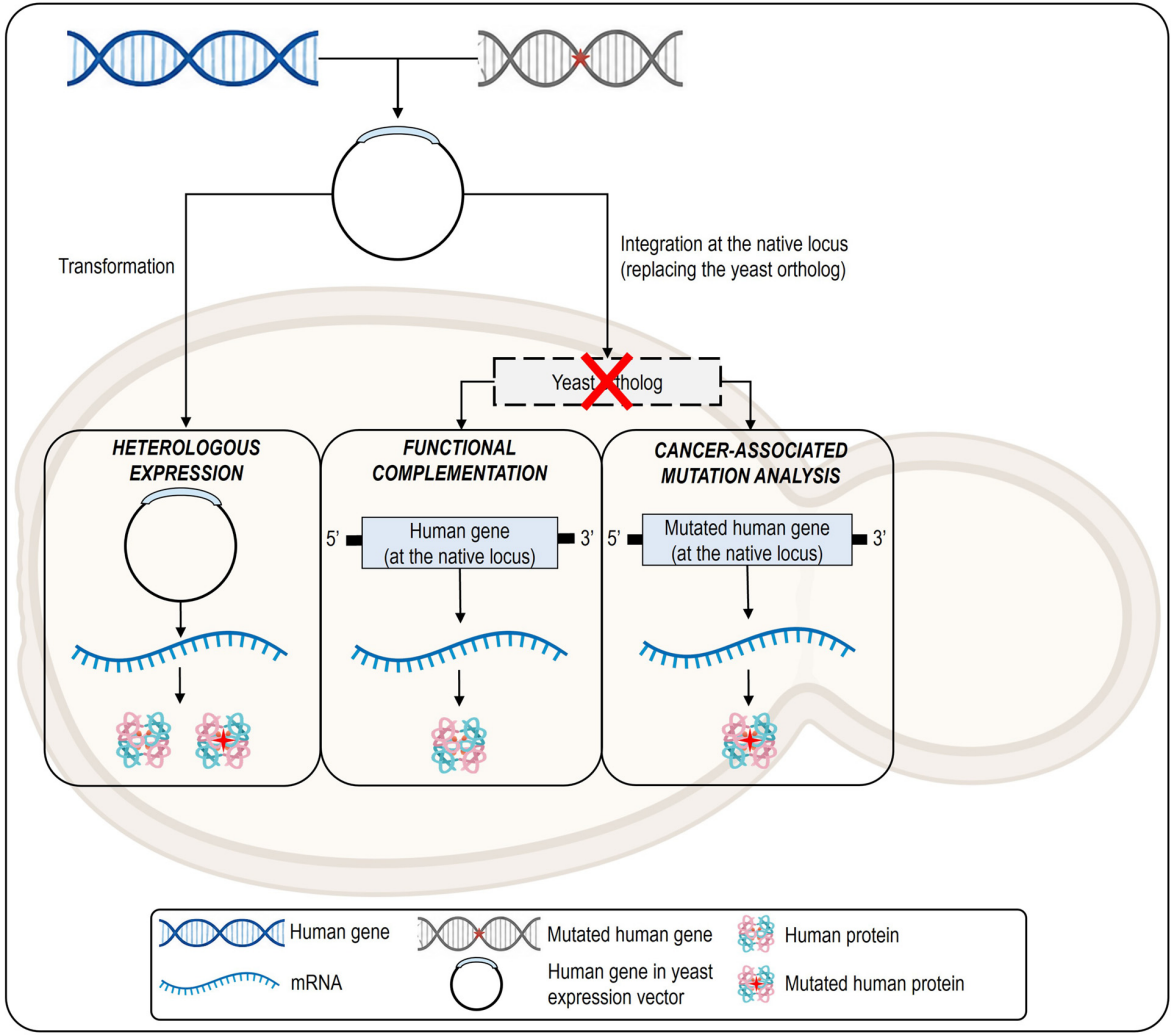
Yeast-based experimental strategies to investigate cancer-associated genes and variants. Schematic representation of **(1)** heterologous expression of human genes in yeast, enabling functional characterization of proteins and associated cellular phenotypes; **(2)** functional complementation assays, in which a yeast gene is replaced by its human ortholog to evaluate conservation of function and rescue of mutant phenotypes; and **(3)***in vivo* analysis of cancer-associated mutations through direct expression of mutated human oncogenes or tumor suppressors, or replacement of the corresponding yeast ortholog, to assess the effects of specific variants on growth, signaling, metabolism, stress responses, and drug sensitivity.

Yeast enables systematic functional interrogation of cancer-associated genes through complementary experimental strategies. Human genes can be heterologously expressed in a genetically tractable background to assess their biochemical and cellular functions in isolation 8. Conversely, conserved yeast orthologs can be replaced with human counterparts to evaluate functional complementation 25, 26. In addition, introduction of cancer-associated variants provides a direct means to quantify their impact on cellular phenotypes, including proliferation control, metabolic regulation, and stress responses 4. These approaches have transformed yeast into a versatile platform for linking genotype to phenotype and for experimentally validating the functional consequences of cancer-associated mutations (Figure 1).

A well-established example of this approach is the analysis of apoptotic regulators. Although yeast lacks endogenous Bcl-2 family members 27, expression of human proapoptotic Bax induces mitochondrial dysfunction and cytochrome c release 28, 29, demonstrating that core elements of cell death regulation can be reconstructed in yeast despite evolutionary divergence 27. This reductionist system has therefore provided important mechanistic insights into mitochondrial apoptosis andthe conserved cellular responses triggered by pro-death signals 30.

Similarly, yeast has been instrumental in the functional dissection of the tumor suppressor p53, a central regulator mutated in more than half of human cancers 31. Heterologously expressed p53 retains transcriptional activity in yeast, enabling quantitative analysis of its response element specificity, cofactor dependence, and mutation-induced functional alterations 32–41. These properties have been extensively exploited to evaluate cancer-associated p53 variants and to reconstruct aspects of its transcriptional network in a simplified system 34, 39, 40, 42. In addition, yeast-based studies have revealed mechanisms of p53-induced cytotoxicity linked to mitochondrial function and oxidative stress, supporting the evolutionary conservation of stress-induced cell death pathways 43–45. Collectively, these studies highlight how yeast can serve not only as a surrogate platform for p53 functional analysis, but also as a predictive system for interpreting clinically relevant mutations.

The absence of endogenous p53 also enables identification of alternative regulatory mechanisms governing p53 target gene activation, supporting the existence of an evolutionarily conserved p53-like DNA-binding activity capable of stimulating transcription in response to genotoxic stress 46. However, the absence of key components of the mammalian p53 regulatory network, including MDM2 and MDM4, limits the reconstruction of the full complexity of p53 signaling 47, 48.

Similar to p53, key components of the PI3K pathway retain their core functions when expressed in *S. cerevisiae*. Reconstitution of the PI3K-Akt pathway in yeast has recapitulated essential regulatory events, including membrane recruitment, lipid signaling, and PTEN-mediated phosphatase activity 33, 34. This system has been widely applied to characterize cancer-associated PTEN mutations and to evaluate pharmacological inhibitors targeting PI3K signaling using growth-based readouts 49–51.

Importantly, these yeast-based assays provide scalable and quantitative platforms for rapidly assessing the pathogenicity of PI3K pathway variants and their responsiveness to targeted therapies. Likewise, yeast RAS signaling provides a robust framework for studying conserved mechanisms of oncogenic activation. Yeast RAS1 and RAS2 proteins share structural and functional properties with human RAS, enabling detailed analysis of post-translational processing, membrane targeting, and downstream signaling via conserved effectors 52–56.

Classical genetic studies in yeast identified key components of this pathway, including adenylate cyclase and Cdc25 57, 58, establishing fundamental principles of RAS regulation that are conserved in higher eukaryotes 59. These systems have further been used to explore cross-species complementation and oncogenic transformation, underscoring the evolutionary conservation of RAS-driven signaling networks 54, 60–62.

Beyond its role in proliferation, yeast RAS signaling has provided insights into cancer-associated metabolic reprogramming and stress adaptation, highlighting conserved links between RAS signaling and cellular metabolism 63–65. In this context, RAS proteins regulate glucose-dependent repression of oxidative metabolism via the cAMP/PKA cascade, a mechanism conceptually analogous to the metabolic shifts observed in tumor cells 66, 67. These findings reinforce the relevance of yeast as a model to investigate the interplay between oncogenic signaling and metabolic rewiring, a central hallmark of cancer progression. Despite these advantages, yeast cannot fully reproduce the complex signaling networks and tissue context that influence RAS-driven tumorigenesis in mammals 48. Consequently, complementary studies in mammalian systems remain essential to assess pathway crosstalk and therapeutic responses in physiologically relevant contexts.

Finally, yeast represents a powerful system for studying cancer-associated alterations in chromatin regulators, including oncogenic histone missense mutations (“oncohistones”) 68. The genetic simplicity of the yeast genome, which contains only two *H3* genes (*HHT1* and *HHT2*) compared with 15 in mammals, and the high evolutionary conservation of histones 68, 69 enable precise functional analysis of chromatin variants and their impact on genome regulation. This has facilitated systematic genetic screening approaches to identify pathways altered by oncohistone mutations and potential therapeutic vulnerabilities 68. Together, these studies demonstrate how yeast chromatin models can reveal conserved epigenetic mechanisms underlying tumorigenesis and support the discovery of novel chromatin-targeted anticancer strategies.

### Cellular metabolic reprogramming

A central paradigm in cancer biology is that malignant transformation is accompanied by extensive metabolic rewiring that sustains the biosynthetic and energetic demands of uncontrolled proliferation and tumor invasiveness 70, 71. Most cancer cells display a characteristic metabolic phenotype defined by aerobic glycolysis, known as the Warburg effect, in which glucose is preferentially converted to lactate rather than being fully oxidized through mitochondrial respiration, even under normoxic conditions 72. This metabolic shift remains dynamically reversible depending on nutrient availability and microenvironmental conditions 73. For instance, in hypoxic tumor regions, such metabolic plasticity supports survival and limits apoptosis 11, 74. However, it remains unclear whether mitochondrial function is preserved or impaired in cancer cells exhibiting the Warburg effect 75.

Interestingly, budding yeast *S. cerevisiae* undergoes a similar metabolic shift under high-glucose conditions, preferentially adopting fermentative metabolism even in the presence of oxygen. This phenomenon, known as the Crabtree effect, channels carbon into biosynthetic pathways that sustain cell growth and proliferation 76.

The Warburg and Crabtree effects are expected to be compared as metabolic analog phenomena because both share phenotypic characteristics, such as a high growth rate accompanied by increased glycolytic flux and fermentation 77. For these reasons, the Crabtree effect is widely used as a model of the Warburg effect 67, 77–80.

However, the two metabolic programs differ in their effects on mitochondrial respiration. In yeast, the Crabtree effect is associated with glucose repression and a concomitant reduction in mitochondrial respiration 81, 82. On the contrary, the Warburg effect is generally not accompanied by decreased basal mitochondrial respiration 83–85; rather, respiration remains active but is uncoupled from ATP synthesis, primarily supporting anabolic processes while ATP production relies predominantly on aerobic glycolysis 86–88.

In particular, in the Warburg effect, glutaminolysis fuels the Krebs cycle to sustain redox balance and anabolic metabolism 89, whereas during the Crabtree effect the Krebs cycle primarily supports amino acid biosynthesis through α -ketoglutarate-derived glutamate 90.

Recently, a *S. cerevisiae* Δ *snf1* mutant has been identified as a yeast model that more closely recapitulates the metabolic features of the Warburg effect. Unlike wild-type cells, which repress mitochondrial respiration under Crabtree-inducing conditions (10% glucose), the Δ *snf1* strain exhibits increased basal respiration while maintaining active fermentation 91, indicating that deletion of *SNF1* overrides the canonical Crabtree response.

*SNF1*, the yeast ortholog of the highly conserved AMP-activated protein kinase (AMPK) family 92, regulates glucose sensing and metabolic adaptation. In mammalian cells, AMPK loss promotes Warburg-like metabolic reprogramming through p53, thereby supporting tumor growth 93. Likewise, deletion of *SNF1* alters glucose sensing via the Snf1p/Hxk2p/Mig1p pathway, leading to increased expression of the high-affinity glucose transporter HXT4 (a functional analog of mammalian GLUT1) instead of the low-affinity transporter HXT1, the major regulator of glycolytic flux during the Crabtree effect 94, 95. This altered glucose uptake supports the simultaneous maintenance of high mitochondrial respiration and fermentation 96. Furthermore, the Δ *snf1* strain displays enhanced fatty acid synthesis 97, consistent with the anabolic phenotype observed in Warburg-like tumor cells 9, 98.

The yeast Δ *snf1* did not carry additional mutations, offering a tractable system to uncover the mechanisms behind the Warburg effect and to support HTS of therapeutic compounds targeting Warburg-like metabolism 96.

#### Conserved regulation of glycolysis and metabolic flux

**Figure 2:**
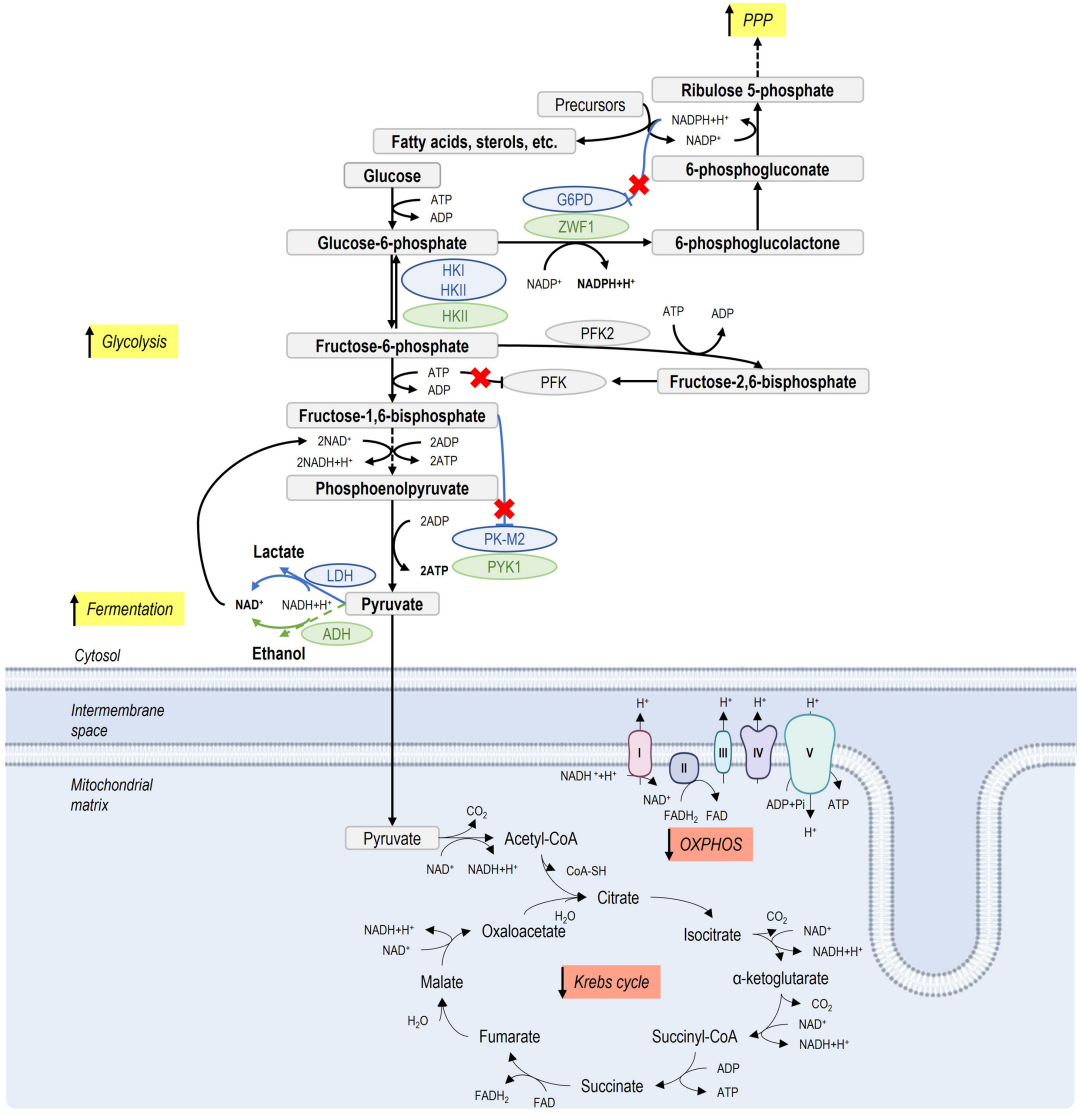
Comparative overview of metabolic rewiring in cancer and yeast cells. Metabolic pathways undergoing similar reprogramming in tumor and yeast cells are depicted. Alterations in glucose uptake, regulation of glycolytic enzymes, and pyruvate fate support enhanced glycolytic flux. Pyruvate is preferentially diverted from mitochondrial oxidation toward lactate production in cancer cells or alcoholic fermentation in yeast, ensuring NAD ${}^{+}$ regeneration and sustaining rapid proliferation. Accumulation of upstream glycolytic intermediates further fuels anabolic pathways, including the pentose phosphate pathway (PPP), to support lipid biosynthesis and biomass production. Core pathways shared between yeast and tumor cells are shown in black, yeast-specific metabolites and enzymes are highlighted in green, and cancer-specific features are shown in blue. Bold labels and thicker arrows indicate predominant metabolic routes. ADH, alcohol dehydrogenase; G6PD, glucose-6-phosphate dehydrogenase; HK, hexokinase; LDH, lactate dehydrogenase; OXPHOS, oxidative phosphorylation; PFK, phosphofructokinase; Pi, inorganic phosphate; PK-M2, M2-isoform of pyruvate kinase; PPP, pentose phosphate pathway; PYK1, pyruvate kinase 1.

At the molecular level, glycolytic reprogramming in both systems is associated with coordinated regulation of key rate-limiting enzymes, including hexokinase (HK), phosphofructokinase (PFK), and pyruvate kinase (PK), as well as increased glucose uptake 66, 67 (**Figure 2**).

In mammals, HK includes four isoforms (I – IV), among which HKI ed HKII are high-affinity isoforms associated with the outer mitochondrial membrane, enabling efficient coupling between glycolysis and ATP supply 66, 99. In *S. cerevisiae*, HKII is the main isoform during fermentation, but it is not associated with the mitochondrial outer membrane due to the absence of the N-terminal targeting region 66. Despite these structural differences, both systems converge on maintaining elevated glycolytic throughput to sustain proliferative metabolism.

Regarding PFK, in both cancer and yeast cells, it shows high activity, characterized by reduced sensitivity to ATP- and citrate-mediated inhibition and increased activation by fructose 2,6-bisphosphate 67, 100. This regulatory configuration promotes sustained carbon flux through glycolysis even under conditions that would normally suppress pathway activity.

PK further exemplifies functional convergence. In cancer cells, a marked predominance of the M2-isoform of PK (PK-M2) has also been reported 101. PK-M2 activity depends on its quaternary structure: the tetramer form is highly active, whereas the dimer is less active. In tumors, PK-M2 is often found in its less active dimeric form, which prevents the binding of its allosteric activator, fructose 1,6-bisphosphate. This partial inhibition promotes the accumulation of upstream glycolytic intermediates, which can be deviated toward anabolic pathways to support rapid cell proliferation 102. In yeast, PYK1 is constitutively expressed during fermentation, while PYK2 is glucose-repressed, collectively ensuring sustained glycolytic flux under fermentative conditions 66 (Figure 2). Together, these observations illustrate how modulation of PK activity represents a conserved strategy to balance energy production with anabolic growth requirements.

In both systems, pyruvate links the regulation of glycolytic enzymes, mitochondrial metabolism and lactate/ethanol production. Limited mitochondrial import of pyruvate and inhibition of the pyruvate dehydrogenase complex (PDH) favor cytosolic accumulation and enhanced activity of lactate dehydrogenase (LDH) in cancer or alcohol dehydrogenase (ADH) in yeast, thereby sustaining NAD ${}^{+}$ regeneration and continuous glycolytic throughput 66, 103 (Figure 2).

Rapidly proliferating cells further rely on anabolic pathways such as the pentose phosphate pathway (PPP) to maintain NADPH homeostasis and support lipid biosynthesis. The rate-limiting enzyme glucose-6-phosphate dehydrogenase (G6PD) is frequently upregulated in cancer and associated with tumor progression, in part due to the loss of feedback inhibition normally exerted by NADPH and fatty acid intermediates. In yeast, its ortholog *ZWF1* is subject to a similar feedback regulation 9. Functional complementation between human *G6PD* and yeast *ZWF1*-deficient strains further highlights the evolutionary conservation of this pathway 104, 105. This system provides a robust platform for HTS of genetic variants and small molecules targeting redox balance and lipid metabolism, with potential translational relevance in cancer.

#### Tme-driven stress adaptation

*S. cerevisiae* provides a tractable platform to model TME-associated stresses, enabling controlled investigation of cellular adaptation to hypoxia, oxidative stress, and nutrient limitation. Through precise manipulation of metabolic and respiratory states, yeast systems recapitulate key survival strategies adopted by tumor cells under microenvironmental constraints 106. The ability to experimentally reproduce these stress conditions in a simplified eukaryotic system makes yeast particularly valuable for dissecting the molecular basis of tumor adaptation and resilience 107. Experimental modulation of mitochondrial activity has shown that repression of oxidative phosphorylation reduces mitochondria-dependent cell death, in part through decreased reactive oxygen species (ROS) accumulation and enhanced glutathione-dependent scavenging. This metabolic configuration promotes survival in rapidly proliferating yeast populations and mirrors adaptive responses observed in cancer cells exposed to hypoxic or nutrient-poor conditions 106, 108. These findings highlight the evolutionary conservation of stress-response programs linking mitochondrial activity, redox balance, and cellular survival under adverse environmental conditions. In parallel, glucose-induced fermentative metabolism in yeast supports sustained ATP production independently of mitochondrial respiration, providing a robust framework to investigate the functional consequences of metabolic rewiring on cell survival 67. These phenotypes are coordinated by conserved nutrient-sensing pathways, including RAS/cAMP-PKA and TOR signaling, which regulate transcriptional programs controlling stress responses and metabolic adaptation 109.

Importantly, yeast-based models may facilitate the identification of conserved stress-adaptation pathways that can be targeted to sensitize cancer cells to metabolic or chemotherapeutic interventions. Nevertheless, because the TME is shaped by dynamic interactions among cancer cells, stromal cells, immune cells, and the extracellular matrix, yeast-based findings should be integrated with more complex experimental models to fully assess their physiological relevance.

### DNA repair mechanisms and cancer-associated variants

DNA repair defects are a major source of genomic instability in cancer and represent key drivers of mutation accumulation and tumor progression 110. The genetic tractability of *S. cerevisiae* enables systematic dissection of conserved DNA repair pathways, including HR, NER, and BER, within a simplified and controllable cellular context 4, 10. Because these pathways are highly conserved across eukaryotes, yeast provides a powerful framework for investigating how defects in genome maintenance contribute to cancer initiation, progression, and therapeutic response. However, the impact of tissue-specific replication stress, chromatin organization, and tumor microenvironmental factors on genome maintenance cannot be fully recapitulated in this simplified system, highlighting the complementary role of mammalian models for translational validation 111, 112.

For example, in yeast, RAD1, RAD5, RAD51, and RAD52 play central and coordinated roles in multiple DNA repair pathways, including NER, HR, and post-replication repair (PRR). *RAD1* encodes a structure-specific endonuclease that, in complex with RAD10, functions in NER by removing DNA lesions caused by ultraviolet radiation and chemical mutagens, processes that are closely linked to genomic instability in cancer cells 113. *RAD5*, on the other hand, is essential for PRR, facilitating error-free DNA damage tolerance through template switching during stalled replication fork progression, and so preventing mutagenic lesion bypass 114, 115. *RAD51* is essential for HR, forming nucleoprotein filaments on single-stranded DNA to mediate homology search and strand invasion during double-strand break repair, while *RAD52* gene product facilitates RAD51 loading and promotes annealing of complementary single-stranded DNA 116. The functional conservation of these proteins has enabled detailed mechanistic insights into how defects in DNA repair pathways contribute to tumorigenesis. Importantly, these studies have established yeast as a foundational system for uncovering conserved principles of genome surveillance and DNA damage tolerance.

A major advantage of yeast-based systems lies in their application to functional genomics and variant interpretation. Human DNA repair gene variants, including those identified in clinically relevant tumor suppressors such as *BRCA1* and *BRCA2*, can be directly expressed in yeast to assess their impact on genome stability, DNA damage sensitivity, protein interactions, and cell viability independently of the complex regulatory networks present in mammalian cells. These approaches provide scalable and cost-effective platforms to distinguish pathogenic mutations from benign variants, thereby supporting clinical variant classification and the development of targeted therapeutic strategies 117.

Beyond canonical DNA repair functions, yeast-based studies have also revealed previously unappreciated roles of tumor suppressors in cellular adaptation. For example, a recent study revealed that the tumor-suppressor protein BRCA2 plays a role in regulating cellular metabolism under stress conditions, extending far beyond its classical function in DNA double-strand break repair through HR. By expressing human *BRCA2* in yeast and performing transcriptomic analyses during acetic-acid-induced regulated cell death, BRCA2 drives a coordinated metabolic shift characterized by increased glycolysis, trehalose and glutathione biosynthesis, alongside suppression of mitochondrial respiration, oxidative phosphorylation, and Krebs cycle activity. This metabolic rewiring is accompanied by activation of the mitochondrial retrograde response and enhanced peroxisomal fatty-acid β -oxidation, suggesting that BRCA2 helps cells adapt to mitochondrial dysfunction by promoting cytosolic energy-producing pathways while simultaneously repressing cell-cycle progression rather than stimulating DNA repair. These findings support a model in which BRCA2 contributes to tumor suppression not only by safeguarding genome integrity but also by orchestrating metabolic stress responses that influence the cellular decision between survival, proliferation arrest, and regulated cell death 118. This example further illustrates the versatility of yeast as a system for uncovering noncanonical functions of cancer-associated genes that may remain difficult to identify in more complex experimental models.

Yeast systems further enable reconstruction of cancer-associated genome instability phenotypes through controlled engineering of metabolic states and chromosomal rearrangements, providing evidence for a mechanistic link between metabolic alterations and genome instability in cancer development 119.

### Autophagy in cancer progression

Autophagy is a conserved cellular recycling process that plays a context-dependent role in cancer, functioning as both a tumor-suppressive and pro-survival mechanism. While it contributes to cellular quality control and genome stability in early stages, established tumors exploit autophagy to sustain growth and survive under stress conditions such as hypoxia, nutrient limitation, and therapeutic pressure. This functional duality has positioned autophagy as a key target for cancer intervention strategies 13. Understanding how autophagy shifts from a protective homeostatic mechanism to a pathway supporting tumor adaptation therefore represents a major challenge in cancer biology and therapeutic development.

*S. cerevisiae* provides a genetically tractable platform to dissect the molecular basis of autophagy and its role in cancer-relevant processes. Approximately 40 autophagy-related genes (*ATGs*) were originally discovered and characterized in yeast, defining the core steps of autophagosome formation, cargo selection, and vesicle trafficking 120, 121. Their human homologs (such as Beclin-1/ATG6, ATG5, ATG7, and LC3/ATG8) play central roles in tumor suppression, tumor metabolism, and therapy resistance 14. This conservation enables reconstruction of autophagic pathways in a simplified system and supports mechanistic analysis of their regulation.

Yeast-based systems have been extensively applied to investigate how autophagy contributes to cellular adaptation under stress conditions that mimic the TME and how inhibiting autophagy might serve as a potential therapeutic strategy for cancers like lung cancer, breast cancer, and pancreatic cancer. For example, it has been demonstrated that starvation inhibited TOR signaling, one of the most frequently dysregulated signaling pathways in cancer, and so activated autophagy, allowing cells to recycle intracellular components to maintain energy homeostasis and viability 122. These studies established fundamental links between nutrient sensing, metabolic stress, and autophagic activation that remain highly relevant for understanding tumor adaptation and therapy resistance.

Moreover, yeast models have been extensively used to functionally characterize cancer-associated mutations in autophagy genes.

Human orthologs can be expressed or used to complement yeast mutants, enabling quantitative assessment of mutation-induced defects in the autophagic pathway. For example, human *BECN1*, the ortholog of yeast *ATG6*/*VPS30*, has been studied in yeast to assess how specific variants affect autophagy initiation, protein-protein interactions within the PI3K complex, and autophagic flux 123. Similarly, cancer-associated mutations in human ATG4B, a cysteine protease required for LC3/ATG8 processing, have been analyzed using yeast complementation assays to determine whether mutations disrupt proteolytic activity or autophagosome maturation 124. Yeast has also been used to evaluate variants in genes such as *ATG5*, *ATG7*, and *ATG16L1*, where functional assays, including GFP-ATG8 processing, alkaline phosphatase (Pho8 Δ 60) activity measurements, and growth under starvation conditions, allow precise quantification of autophagic flux and identification of defects caused by patient-derived mutations 125, 126. These approaches are particularly valuable because yeast lacks the genetic redundancy and complex signaling networks present in mammalian cells, enabling clearer interpretation of how specific mutations alter autophagy machinery.

Yeast is also widely used for chemical and genetic screening to identify modulators of autophagy, some of which are being investigated as potential anticancer agents. Using assays such as GFP- ATG8 processing, Pho8 Δ 60 activity, or growth under nutrient starvation, researchers screened large compound libraries to determine whether specific molecules stimulate or inhibit autophagy at different stages of the pathway 127.

For example, the discovery of rapamycin as an inhibitor of the TOR signaling pathway in yeast provided the first pharmacological evidence that TOR negatively regulates autophagy, a finding later translated to mammalian systems where mTOR inhibition induces autophagy and influences cancer cell metabolism 122. Conversely, yeast screens have also helped identify compounds such as 3-methyladenine (3-MA) and wortmannin, which inhibit class III PI3Kactivity and thereby block autophagosome formation, as well as chloroquine and hydroxychloroquine, which impair lysosomal/vacuolar degradation and inhibit late-stage autophagy 121, 128–130. Some natural compounds, including resveratrol and spermidine, have also been shown in yeast models to promote autophagy through nutrient-sensing pathways, effects that are being investigated for their potential roles in cancer prevention or therapy 131.

Overall, discoveries made through yeast-based chemical screens often provide a starting point for developing anticancer strategies aimed at either enhancing autophagic cell death or sensitizing tumor cells to chemotherapy by inhibiting cytoprotective autophagy. However, given the highly context-dependent biological consequences of autophagy modulation in cancer 132, 133, the efficacy, bioavailability, and selectivity of these compounds ultimately need to be evaluated in multicellular experimental systems.

## YEAST-BASED PLATFORMS FOR ANTICANCER DRUG DISCOVERY AND DEVELOPMENT

Over the past decades, cancer drug discovery has shifted from conventional cytotoxic approaches to precision medicine based on molecularly targeted therapies. However, the identification of effective and safe treatments remains limited by tumor heterogeneity, adaptive resistance mechanisms, and the complexity of the TME, all of which contribute to variable therapeutic responses 134–139. In addition, the high cost and extended timelines associated with oncology drug development continue to represent major bottlenecks 140. These challenges have intensified the need for experimentally tractable and scalable model systems capable of accelerating target validation, drug screening, and mechanistic characterization of therapeutic responses.

In this context, *S. cerevisiae* offers lower experimental costs, rapid growth, and unparalleled genetic tractability, enabling systematic functional interrogation of drug targets at genome scale 10. However, because drug uptake, metabolism, and pharmacological responses differ substantially between yeast and human cells, candidate compounds identified in yeast require subsequent validation in mammalian models.

First of all, yeast-based HTS has emerged as a powerful tool for the rapid identification of potential anticancer compounds that target essential cancer-related mechanisms such as DNA repair, apoptosis, and cell cycle regulation 17. Yeast-based HTS platforms utilize robotic liquid handling, automated growth monitoring such as optical density or luminescence, and large-scale mutant libraries to evaluate compound effects across thousands of strains or conditions in parallel. These HTS systems are particularly valuable because they enable screening against defined genetic backgrounds, such as specific gene deletions or overexpression constructs, to uncover genotype-dependent drug responses 141. This scalability provides a major advantage over more complex screening platforms, where genome-wide functional analyses remain considerably more expensive.

These systems have been effectively used to dissect key oncogenic pathways. Yeast screens targeting TOR signaling, for instance, have uncovered small molecules that modulate pathway activity and enhance the efficacy of known inhibitors while maintaining low intrinsic toxicity 142. Such studies illustrate how yeast can facilitate the discovery of pathway-specific modulators and support rational combination strategies aimed at overcoming resistance mechanisms in tumors.

A notable example of yeast-based HTS applied to cancer metabolic reprogramming and drug discovery has been the screening performed on more than 200000 compounds using a *S. cerevisiae* model carrying defects in succinate dehydrogenase (SDH), which molecularly recapitulates human SDH-mutated paraganglioma 143. The study identified several compounds selectively toxic toward cells with impaired Krebs cycle function, including inhibitors of yeast ADH. Since ADH is required to regenerate NAD ${}^{+}$ in glycolytic yeast cells lacking functional mitochondrial metabolism, the authors proposed LDH as the analogous metabolic vulnerability in human *SDH*-deficient tumors. Consistently, *SDH*-deficient human cells displayed increased sensitivity to pharmacological inhibition of LDH, highlighting this pathway as a potential therapeutic target in paraganglioma 143. Yeast chemogenomic approaches have been used to dissect genetic determinants of resistance to carcinogen-induced DNA damage. A genome-wide screen in *S. cerevisiae* using barcoded deletion libraries and human metabolic enzymes identified conserved gene networks involved in DNA repair and cellular metabolism that modulate tolerance to the activated dietary carcinogenic heterocyclic aromatic amine 2-amino-3-methylimidazo[4,5-f] quinoline (IQ). A subset of these candidate resistance genes was validated using growth curves, competitive growth assays, and viability staining, revealing major gene clusters involved in ribosomal structure, DNA damage response and repair, nitrogen metabolism, and protein modification. Notably, many validated yeast resistance genes had human orthologs associated with colon cancer risk, including *NTHL1* and *RAD18*, suggesting that conserved pathways mediating tolerance to IQ-induced DNA damage could be relevant to carcinogenesis 144.

In parallel, classical studies using yeast have been instrumental in validating key anticancer targets, such as topoisomerase I (TOP1), and in supporting the development of clinically relevant inhibitors, including camptothecin derivatives. In *S. cerevisiae*, *TOP1* deletion confers resistance to the drug, confirming its target specificity 145. Yeast-based assays have also enabled the identification of resistance-conferring mutations and the screening of camptothecin derivatives, supporting the development of clinically relevant analogs such as topotecan and irinotecan 146, 147.

More recently, functional genomic approaches in yeast have been applied to characterize responses to proteasome inhibitors, such as bortezomib, and to identify mutations that modulate drug sensitivity, providing mechanistic insight into resistance pathways and informing strategies to overcome resistance in clinical settings 148–150. Bortezomib targets the 26S proteasome, a multi-subunit complex responsible for degrading ubiquitinated proteins, and is widely used in the treatment of multiple myeloma and other hematologic malignancies. In yeast, genome-wide deletion libraries and mutagenesis screens revealed that mutations in the proteasomal β 5 subunit (*PUP*1 in yeast, the ortholog of human *PSMB5*), which harbors the drug’s binding site, confer resistance by reducing bortezomib binding affinity while retaining partial catalytic activity 148. Additionally, alterations in regulatory subunits and associated chaperones were found to modulate proteasome assembly and function, influencing cellular tolerance to proteasome inhibition 149, 150.

Yeast has long served as a powerful model for studying synthetic lethality, a genetic interaction in which the simultaneous disruption of two genes results in cell death, whereas loss of either gene alone is compatible with viability. This concept has strong therapeutic relevance in oncology, where it is exploited to selectively target tumors harboring specific genetic defects, such as the use of PARP inhibitors in *BRCA1*- or *BRCA2*-deficient cancers 151. In yeast-based systems, chemical libraries can be screened against defined gene deletion strains to identify compounds that are selectively toxic to cells lacking particular genes, thereby modeling tumor-specific vulnerabilities. Another complementary strategy involves heterologous overexpression of a human protein of interest in yeast, which often produces a measurable phenotype such as impaired growth. Compounds that reverse this phenotype by inhibiting the overexpressed protein can then be identified as candidate therapeutics with defined molecular targets. For example, expression of human PARP1 in yeast induces growth defects that are alleviated by established PARP1 inhibitors, validating the system for drug discovery 152. A similar approach has been applied to tankyrase 1, another member of the PARP family implicated in BRCA-associated cancers, where HTS identified a flavone compound capable of restoring yeast growth and subsequently demonstrating efficacy in mammalian cell models 153. Beyond small-molecule discovery, emerging yeast-based platforms are being developed to support immunotherapy research. Engineered yeast displaying human tumor antigens on their surface can be used as scalable systems to study immune recognition and to screen chimeric antigen receptor (CAR) designs, offering a more efficient alternative to traditional cancer cell models 154. The platform has the potential to accelerate the development and optimization of safer and more effective CAR T cell therapies, particularly by improving understanding of antigen-specific responses 154. In particular, Deichmann et al. demonstrated that yeast-based antigen presentation systems can effectively simulate CD19+ cancer cells with precise on-demand antigen densities that control the activational state of CAR T cells 154. These findings highlight the utility of yeast platforms for dissecting antigen-specific immune responses and for identifying CAR architectures with improved specificity and reduced risk of off-target toxicity. These innovative applications further expand the translational potential of yeast, positioning it not only as a model for cancer biology but also as a versatile platform for next-generation therapeutic development.

## CONCLUSION AND FUTURE PERSPECTIVES

**Figure 3:**
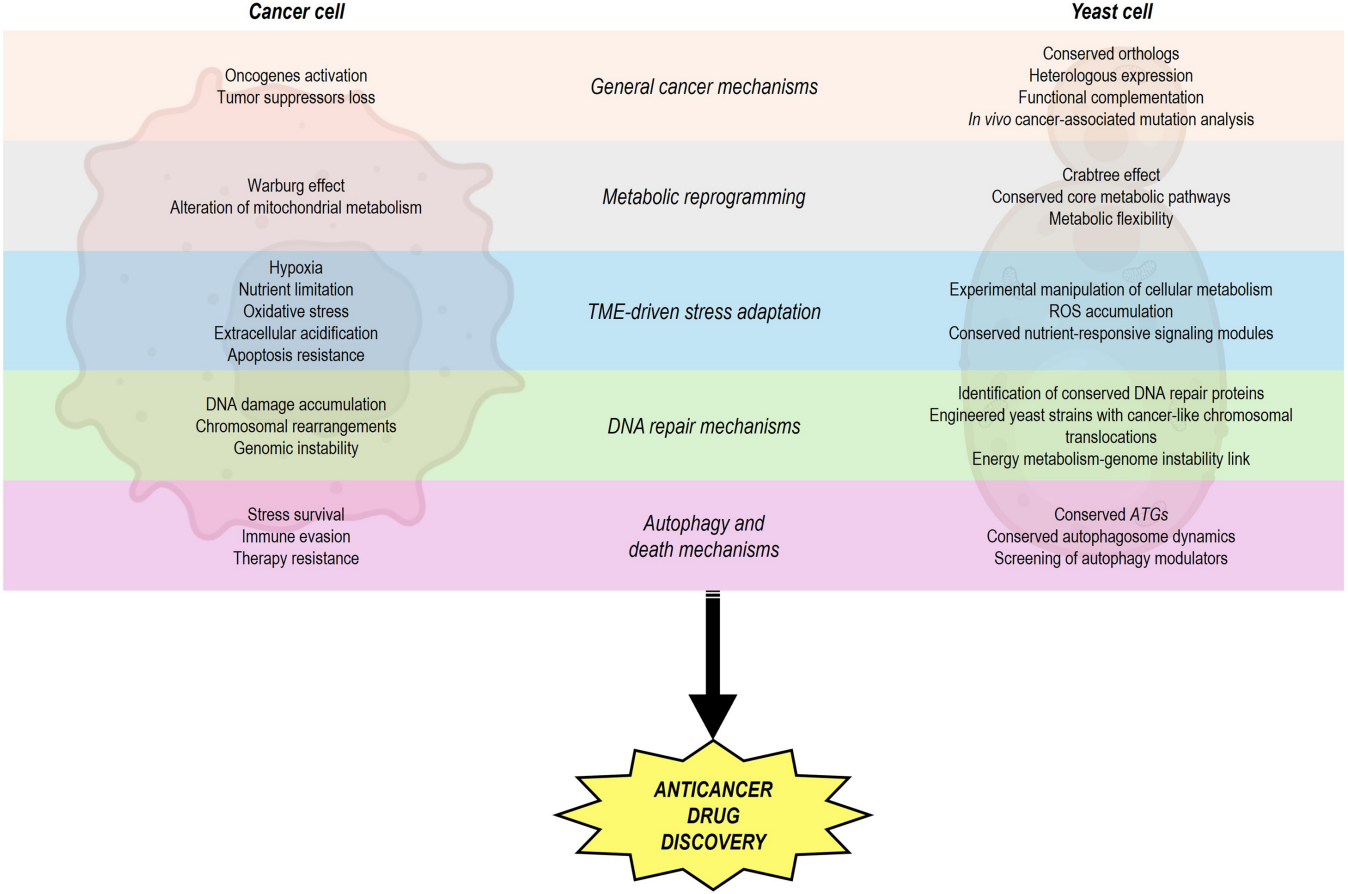
Yeast as a platform to model key features of cancer cells. The cancer cell highlights major cancer-associated processes, including oncogenic signaling pathways, metabolic reprogramming, adaptation to microenvironmental stress, DNA repair alterations, and autophagy. The yeast cell illustrates the corresponding features that make it a valuable experimental system to investigate these processes, such as genetic tractability, conservation of core pathways, ease of metabolic manipulation, and suitability for high-throughput functional and chemical screening. ATGs, autophagy-related genes; ROS, reactive oxygen species; TME, tumor microenvironment.

The evidence discussed in this review highlights the unique contribution of the budding yeast *S. cerevisiae* to advancing our understanding of cancer biology and supporting anticancer drug discovery. The remarkable conservation of core cellular processes, including oncogenic signaling, metabolic regulation, adaptation to microenvironmental stress, DNA repair, and autophagy, enables the reconstruction and systematic dissection of cancer-associated mechanisms within a genetically tractable eukaryotic model 27, 30, 43, 53, 66, 67, 117, 130. This experimental simplicity provides a major advantage, allowing precise functional analysis of genes, pathways, and variants that are often difficult to study in more complex mammalian systems (Figure 3).

Beyond pathway reconstruction, yeast has proven particularly valuable for the functional interpretation of cancer-associated variants and for the investigation of genotype-specific responses to therapeutic agents 10, 17, 155–157. Importantly, several discoveries initially made in yeast, such as the characterization of conserved signaling pathways and the validation of pharmacological targets, have successfully translated into mammalian systems and clinical applications, underscoring the predictive value of this model 155, 158.

Although yeast is undoubtedly a powerful metabolic model, caution is required when translating these findings to human oncology. As a unicellular organism, yeast cannot recapitulate several key hallmarks of human cancer, including interactions with the immune system, complex proliferative signaling networks, multicellularity and tissue architecture underlying the TME, cell-cell communication associated with invasion and metastasis, and angiogenesis 96. Consequently, combining yeast with mammalian models, patient-derived systems, organoids, and computational approaches will likely provide the most informative strategy for translating mechanistic discoveries into clinically relevant insights.

Looking forward, several emerging directions are expected to further expand the impact of yeast in cancer research. Advances in synthetic biology and genome engineering will facilitate the construction of more sophisticated humanized yeast models, capable of recapitulating complex oncogenic pathways and protein interaction networks with higher fidelity 6, 16, 26, 159. In parallel, the application of machine learning to large-scale yeast datasets holds promise for improving the prediction of variant pathogenicity, drug responses, and synthetic lethal interactions 160, 161.

In conclusion, the integration of emerging technologies with yeast-based HTS, chemogenomics, and systems biology approaches is expected to further strengthen the role of yeast as a versatile discovery engine, bridging fundamental cancer biology with translational research and accelerating the development of precision oncology and next-generation anticancer therapies.

## CONFLICT OF INTEREST

The authors declare no conflicts of interest.

## ABBREVIATIONS

3-MA – 3-methyladenine

ADH – alcohol dehydrogenase

AMPK – AMP-activated protein kinase

ATGs – autophagy-related genes

BER – base excision repair

CAR – chimeric antigen receptor

G6PD – glucose-6-phosphate dehydrogenase

HK – hexokinase

HR – homologous recombination

HTS – high-throughput screening

IQ – 2-amino-3-methylimidazo[4,5-f] quinoline

LDH – lactate dehydrogenase

NER – nucleotide excision repair

OXPHOS – oxidative phosphorylation

PDH – pyruvate dehydrogenase complex

PFK – phosphofructokinase

Pi – inorganic phosphate

PK – pyruvate kinase

PK-M2 – M2-isoform of pyruvate kinase

PPP – pentose phosphate pathway

PRR – post-replication repair

PYK1 – pyruvate kinase 1

PYK2 – pyruvate kinase 2

ROS – reactive oxygen species

SDH – succinate dehydrogenase

TME – tumor microenvironment

TOP1 – topoisomerase I
